# A transvaginal ultrasound-based diagnostic calculator for uterus post-cesarean scar defect

**DOI:** 10.1186/s12905-023-02715-3

**Published:** 2023-10-27

**Authors:** Zahra Allameh, Safoura Rouholamin, Sina Rasti, Atoosa Adibi, Zahra Foroughi, Maryam Goharian, Mehrdad Rabiee Rad, Ghazal Ghasempour Dabaghi

**Affiliations:** 1 Department of Obstetrics and Gynecology, Isfahan University of Medical Sciences, Ostad Motahari St., Felezi Bridge, Isfahan, Iran; 2 Department of Radiology, Isfahan University of Medical Sciences, Isfahan, Iran; 3 School of Medicine, Isfahan University of Medical Sciences, Isfahan, Iran

**Keywords:** Abnormal uterine bleeding, Cesarean section, Cesarean scar defect, Transvaginal ultrasound, Hysteroscopy

## Abstract

**Background:**

A cesarean scar defect (CSD) is incomplete healing of the myometrium at the site of a prior cesarean section (CS), complicating more than half of all cesarean sections. While transvaginal ultrasound (TVU) is the most common modality for diagnosing this defect, hysteroscopy remains the gold standard. We aimed to develop an efficient diagnostic tool for CSD among women with abnormal uterine bleeding (AUB) by integrating TVU findings and participants’ demographic features.

**Methods:**

A single-center cross-sectional study was conducted on 100 premenopausal and non-pregnant women with a history of CS complaining of AUB without a known systemic or structural etiology. Each participant underwent a hysteroscopy followed by a TVU the next day. The defect dimensions in TVU, patients’ age, and the number of previous CSs were integrated into a binary logistic regression model to evaluate their predictive ability for a hysteroscopy-confirmed CSD.

**Results:**

Hysteroscopy identified 74 (74%) participants with CSD. The variables age, the number of CSs, defect length, and defect width significantly contributed to the logistic regression model to diagnose CSD with odds ratios of 9.7, 0.7, 2.6, and 1.7, respectively. The developed model exhibited accuracy, sensitivity, and specificity of 88.00%, 91.89%, and 76.92%, respectively. The area under the receiver operating curve was 0.955 (P-value < 0.001).

**Conclusion:**

Among non-pregnant women suspected of CSD due to AUB, looking at age, the number of previous CSs, and TVU-based defect width and length can efficiently rule CSD out.

## Background

With the rising rate of cesarean deliveries in recent decades, [1] the complications associated with cesarean section (CS) have become increasingly relevant. Among these complications, inadequate anterior uterine wall scar tissue healing has been linked to various known consequences. These include cesarean scar pregnancy, morbid placental adherence, postmenstrual spotting, dysmenorrhea, dyspareunia, pelvic pain, secondary infertility, and scar rupture/dehiscence [2].

Cesarean scar defect (CSD), also known as isthmocele and cesarean scar niche, refers to incomplete myometrial thickness at the site of a previous CS. This defect usually remains limited to the uterine cavity, while an extension to the skin has been reported as the most severe variety, causing a uterocutaneous fistula [3]. Although the prevalence of CSD remains unclear, Stegwee et al. likely conducted the most robust study to date to evaluate it. They reported that 71% of women were diagnosed with CSD by transvaginal ultrasound (TVU) 3 months after their first CS [4]. When it comes to women who present with abnormal uterine bleeding (AUB), this prevalence is expectedly much higher; reportedly, 76.4% of those who are referred for gynecological imaging with evidence of CSD suffer from AUB [5]. Letting alone the critical consequences like scar pregnancy, dehiscence, or infertility, AUB and niche-related pains impose a significant quality of life deterioration [2]. Such a high prevalence and negative health impact of this condition indicate the need to develop screening and diagnostic standards for detecting CSD and preventing its potentially adverse outcomes.

Various diagnostic modalities, including magnetic resonance imaging, hysteroscopy, hysterography, and transabdominal or TVU with or without saline/gel instillation, have been employed to detect CSD [6]. However, hysteroscopy, considered the gold standard, is a high-cost and invasive procedure and may not be readily accessible in all treatment centers [7]. Additionally, it carries risks such as anesthesia-related complications and, in rare cases, uterus perforation, a potentially life-threatening consequence [8].

In contrast, TVU is a rapid, minimally invasive, and cost-effective tool that can conveniently be performed in an outpatient setting [9]. Moreover, the current literature introduces patient characteristics like age and the number of previous CSs as potential factors affecting CSD morphology [10].

To the extent of our knowledge, no other research has developed a diagnosis calculator based on an individual’s history and TVU parameters. This study aims to generate such a model and evaluate its diagnostic efficacy compared to the gold standard (hysteroscopy) for patients with a history of CS experiencing AUB. By examining the diagnostic value of this model, we can assess its potential as a screening or primary method for CSD detection in a safe and accessible manner.

## Methods

### Sampling and study design

This cross-sectional study was conducted at Shahid Beheshti Hospital, Isfahan, Iran, over consecutive referrals between December 2020 and November 2021. The hospital serves as the main tertiary-care center for obstetric and gynecologic conditions, ensuring a diverse patient population for comprehensive evaluation. After the Ethics Committee of Isfahan University of Medical Sciences approved the study protocol (IR.MUI.MED.REC.1397.044), spoken and written informed consent was obtained from all eligible participants.

Inclusion criteria comprised non-pregnant female patients aged 25–45 years with a history of low-transverse CS, no previous CS less than six months before the study, AUB at reproductive age with unproven etiology, normal uterine anatomy, and consent to take part in all study procedures.

To detect any conditions that may provoke AUB or distort uterine anatomy, a thorough physical examination was conducted by a single expert gynecologist. Transabdominal ultrasonography was performed to exclude uterine or cervical abnormalities. Laboratory test results were assessed, including β human chorionic gonadotropin, T4, thyroid-stimulating hormone, prolactin, activated partial thromboplastin time, prothrombin time, and thrombin time.

The study did not include participants showing any abnormality in the screenings above, ensuring a homogeneous sample. Whether a participant could not cooperate during hysteroscopy due to reasons like anxiety or pain, she was excluded from the study. Also, we excluded participants with a history of more than three CSs because of their small population (n = 2), achieving a sample size of 100.

### Hysteroscopy

All participants underwent office hysteroscopy without general anesthesia during the follicular phase of their menstrual cycle. This was determined according to their last menstrual period date. To ensure patient comfort, a single dose of acetaminophen tablet (500 mg) was administered to each patient one hour before the procedure.

The procedure was conducted with participants in a lithotomy position, ensuring an empty urinary bladder. After inserting a sterile speculum, the cervix was thoroughly disinfected using a 4% chlorhexidine solution. Subsequently, a CH 8 Foley catheter was carefully introduced into the uterine cavity via the cervix, and its balloon was inflated with five mL of saline to stabilize the internal cervical os.

A rigid 2.7 mm hysteroscope (Richard Wolf, Knittlingen, Germany) was used to visualize the uterine cavity, employing an isotonic sodium chloride distention medium. As precise diagnostic criteria for identifying CSD were not found in the existing literature, any observed defect or indentation at the scar site was considered indicative of a CSD.

After the procedure and evacuation of the distension medium, participants were observed for 2 h and discharged in full health.

### Transvaginal ultrasound

A day following the hysteroscopy procedure, a TVU was performed by a skilled radiologist, who remained blind to the hysteroscopy results. TVU examinations were conducted using Samsung H60 EV (Samsung Medison CO., Ltd, Seoul, Korea) equipped with a 4–9 MHz vaginal probe.

During the TVU assessment, the radiologist focused on identifying the characteristic features of a CSD within the anterior uterine wall. The scar niche typically appears as a conoid region of lucency, exhibiting an apex and a base. The apex is at the innermost part of the remaining myometrium, while the base aligns with the adjacent endometrial/cervical surface.

**Fig. 1:**
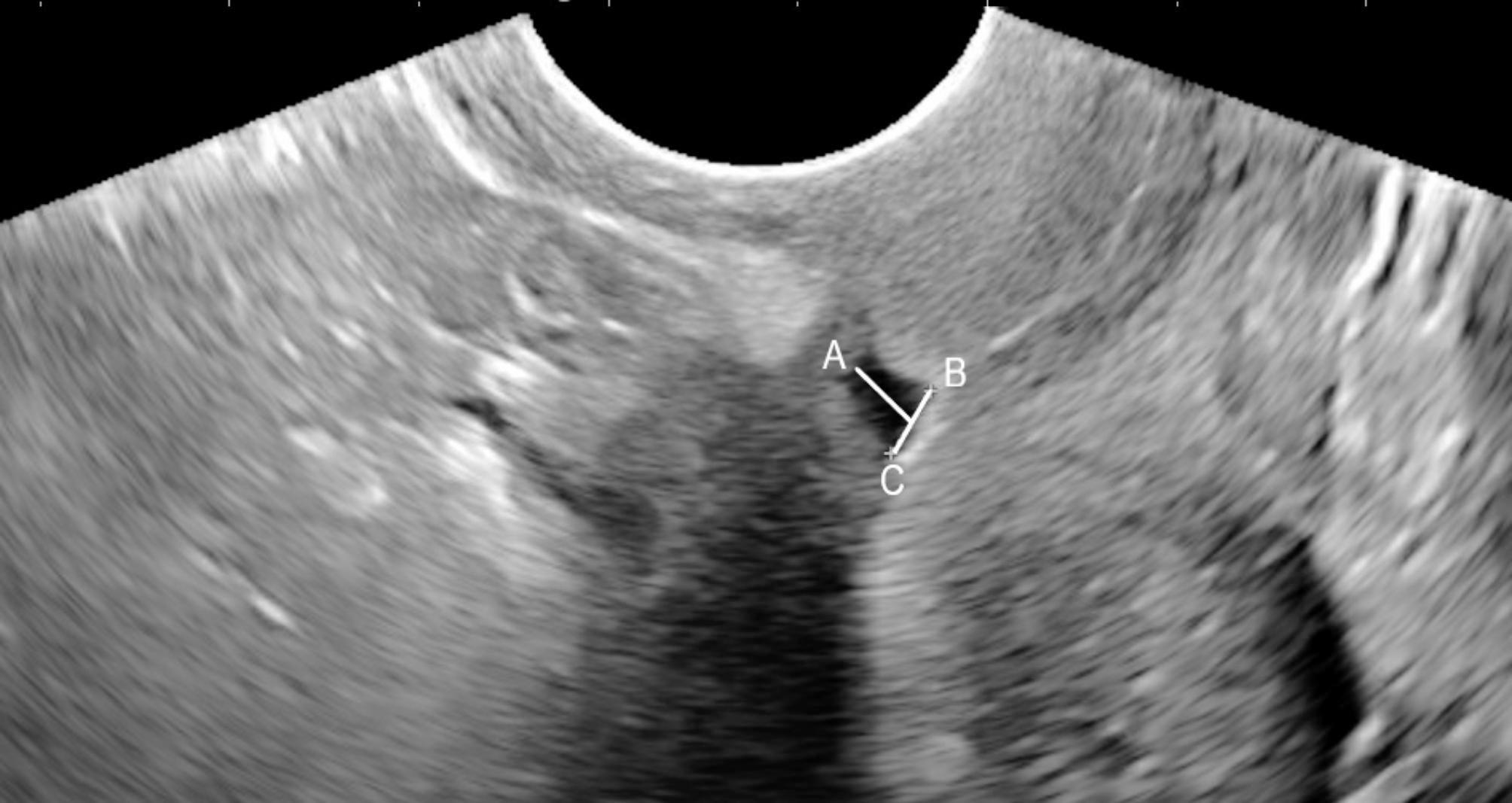
Transvaginal Ultrasonographic Appearance of a Cesarean Scar Defect in Sagittal View. The letter A represents the apex, while the BC line represents the base. The defect’s length is equal to the BC line’s length; the defect’s depth is defined as the distance between the apex and the middle of the BC line

Not any defect in the location of a previous CS is considered a CSD. A depth of at least 2 mm is usually required to label a defect as CSD in TVU [11]. To quantify a defect’s dimensions, specific parameters were defined. The defect width was determined as the maximum diameter of the niche base in the transverse plane. The length was measured in the sagittal plane using the same criterion [12]. The depth was quantified as the minimum distance between the apex and the base (Fig. 1).

### Statistical analysis

Variables, including age, the number of previous CSs, the presence of an intrauterine indent in hysteroscopy, and defect dimensions (length, width, and depth), were recorded for each patient. For further analysis, the data were entered into the IBM SPSS for Windows software package (v.26, SPSS Inc., Chicago, IL, USA).

Quantitative variables were reported as mean ± standard deviation (SD) or median (interquartile range), while categorical data were expressed as percentages and counts. Variable distribution normality was assessed by Kolmogorov-Smirnov (n ≥ 30) or Shapiro-Wilk (n < 30) test. Between-group comparisons were conducted using the independent samples t-test (for normally distributed data) or the Mann-Whitney U test (for non-normally distributed data). Categorical variables were compared using the χ2 test. Statistical significance was set at P < 0.05 (two-tailed).

A binary logistic regression model was employed to assess whether recorded defect dimensions in TVU, along with age and the number of previous CSs, could predict hysteroscopy results. A backward stepwise method based on Wald statistics was employed to exclude variables with no significant effect (α-type error probability > 0.1 was set for this purpose). Adjusted odds ratios (ORs) with 95% confidence intervals (CIs) were reported for each predictor. The post hoc power of the final regression model was assessed using the Hosmer-Lemeshow goodness-of-fit test. Receiver operating characteristics (ROC) curve analysis was conducted using MedCalc Statistical Software version 20.218 (MedCalc Software Ltd, Ostend, Belgium) to estimate the optimal cutoff values for niche dimensions that could predict CSD in hysteroscopy. This cutoff was chosen as the value with the maximum Youden Index.

The diagnostic values of the regression model and defect dimension cutoffs were reported in terms of accuracy, sensitivity, specificity, positive predictive value (PPV), and negative predictive value (NPV). Positive and negative likelihood ratios were also reported to aid in comparing the predictive abilities of the dimensions. The areas under the ROC curves (AUROCCs) of the three dimensions were compared using DeLong et al.‘s method to determine if any dimension outperformed the others in diagnosing CSD [13].

## Results

We achieved a final sample (N = 100) with a median age of 34.50 (IQR: 28.25-38.00). Regarding the number of previous CSs, 35 (35%), 33 (33%), and 32 (32%) experienced one, two, and three surgery episodes, respectively. Table 1 summarizes demographic findings. It was observed that none of the quantitative variables exhibited a normal distribution, either overall or within the subcategories.

**Table 1:** Demographic Data

demographic	CSD in Hysteroscopy		
	no N = 26 [26.0%]	yes N = 74 [74.0%]	*P* value
Participants’ Age—year	31.5 (27.0–38.0)	35.0 (29.0–37.0)	0.584
Defect Length—mm	1.5 (1.0–2.0)	7.0 (4.0–9.0)	< 0.001^a^
Defect Width—mm	2.0 (2.0–3.0)	7.0 (4.0–11.0)	< 0.001^a^
Defect Depth—mm	0.3 (0.2–0.4)	4.0 (2.1–10.0)	< 0.001^a^
Patients with one previous CS	15 (57.7%)	20 (27%)	0.007^a^
Patients with two previous CSs	8 (30.8%)	25 (33.8%)	
Patients with three previous CSs	3 (11.5%)	29 (39.2%)	

^a^significant *P* value (< 0.05)

Quantitative variables are reported as median (interquartile range); Categorical variables are reported as count (%)

CS: cesarean section, CSD: cesarean scar defect, TVU: transvaginal ultrasonography

Table 2 presents the regression model results, which explored the association between variables and hysteroscopy-approved CSD. The Hosmer-Lemeshow test demonstrated this model to be well-fitted (*P* = 0.986). Age, the number of prior CSs, and the defect’s length and width were significantly associated with hysteroscopy-endorsed CSD. Defect depth showed no significant contribution to the model and, therefore, was excluded during the backward stepwise regression analysis. While the length, width, and number of previous CSs exhibited positive correlations with hysteroscopy-approved CSD, age demonstrated a negative correlation. These findings indicate that age and the number of previous CSs can influence the likelihood of CSD, and TVU can serve as an efficient diagnostic tool in predicting hysteroscopy results.

**Table 2:** Binary Logistic Regression Results for Predicting Hysteroscopy-Approved CSD

variable	B coefficient	S.E. of B	Adjusted OR (95%CI)	*P* value
Patient’s Age—year	-0.368	0.143	0.692 (0.523–0.916)	0.010^a^
Defect Length—mm	0.962	0.342	2.616 (1.337–5.119)	0.005^a^
Defect Width—mm	0.545	0.283	1.724 (0.990–3.003)	0.054
Number of Previous CSs	2.269	0.954	9.666 (1.491–62.655)	0.017 ^a^
Constant	4.123	2.810		

^a^significant *P* value (< 0.05)

CS: cesarean section, CSD: cesarean scar defect, S.E.: standard error, TVU: transvaginal ultrasonography

The regression model exhibited an adequate predictive accuracy for hysteroscopy results, with an overall accuracy of 88.00% (CI: [81.63, 94.37]). The model also showed high sensitivity (91.89%, CI: [86.54, 97.24]) and PPV (91.89%, CI: [86.54, 97.24]). However, the specificity (76.92%, CI: [68.67, 85.18]) and NPV (76.92%, CI: [68.67, 85.18]) were relatively low. The model’s AUROCC was 0.955, CI: (0.895, 0.986).

**Fig. 2:**
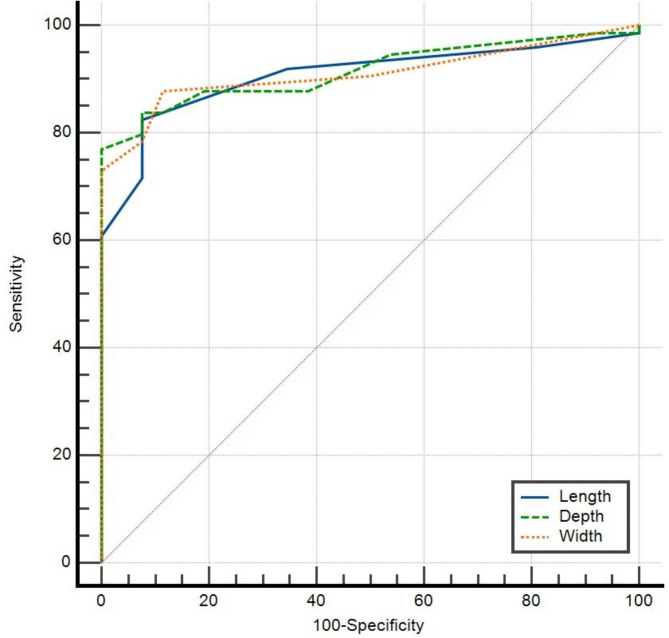
ROC curve of Niche Dimensions for Diagnosing CSD by TVU. CSD: cesarean scar defect, ROC: receiver operating characteristics, TVU: transvaginal ultrasound

Table 3 presents the ROC curve analysis results for the defect dimensions. The AUROCC was calculated to assess the predictive capabilities of different dimensions. However, no significant differences were observed between the AUROCCs, indicating that all dimensions had similar diagnostic performance in predicting CSD (Fig. 2).

**Table 3:** Predictive Function of Each Defect Dimension to Predict a Hysteroscopy-Endorsed Cesarean Scar Defect

Predicting variable	AUROCC (95%CI)	Optimal Cutoff	Sensitivity (95%CI)	Specificity (95%CI)	PPV (95%CI)	NPV (95%CI)	PLR	NLR
Defect Width—mm	0.910 (0.835–0.958)	2 mm	87.84 (78.2–94.3)	88.46 (69.8–97.6)	95.6 (88.2–98.4)	71.9 (57.7–82.7)	7.61	0.14
Defect Length—mm	0.907 (0.833–0.956)	3 mm	82.43 (71.8–90.3)	92.31 (74.9–99.1)	96.8 (88.9–99.1)	64.9 (52.7–75.4)	10.72	0.19
Defect Depth—mm	0.923 (0.852–0.967)	2 mm	77.03 (65.8–86.0)	100.00 (86.8–100.0)	100.0	60.5 (50.2–69.9)	N/C^a^	0.23

^a^ not calculable

AUROCC: area under the receiver operating characteristics curve, NLR: negative likelihood ratio, NPV: negative predictive value, PLR: positive likelihood ratio, PPV: positive predictive value, CI: confidence interval

## Discussion

The escalating rates of cesarean deliveries, the high likelihood of CSD following the procedure, and the potential consequences of this defect necessitate the development of a cost-effective diagnostic method for its detection. Hysteroscopy, considered by some as the gold standard, is an invasive and costly procedure that limits its widespread use. However, this study highlights the significance of age, the number of previous CSs, and TVU results as predictors of hysteroscopy-approved CSD. The devised regression model in this study exhibited excellent sensitivity, indicating its potential as a valuable tool for identifying patients without CSD. However, clinicians should take note of the slightly lower specificity, which may cause false-positive diagnoses. Furthermore, the findings suggest that all defect dimensions (length, width, and depth) have similar diagnostic capabilities in predicting CSD.

TVU has been widely recognized for its accuracy in detecting structural pathologies related to AUB, such as endometrial polyps, hyperplasia, and submucosal fibroids [14]. Researchers recommend TVU as the initial diagnostic step to assess potential structural sources of AUB. They suggest that if TVU shows no abnormalities, further assessment may not be necessary [9, 15].

Numerous studies support TVU as the modality of choice to diagnose CSD [16]. In a retrospective study on 13 non-pregnant individuals with hysteroscopy-confirmed CSD, TVU could detect the defect in 12 [17]. Fabres et al. reported a 100% correlation between TVU and hysteroscopic findings in a retrospective study of 32 people [9]. A survey of 70 asymptomatic subjects found that transvaginal ultrasound had 100% sensitivity and specificity [18]. The current study’s TVU-based model demonstrated a sensitivity of 91% for diagnosing CSD among participants with AUB, making it a robust tool for ruling out this condition. Although our model’s diagnostic performance seems inferior to those of other studies, it is built upon a larger sample size of homogeneous symptomatic participants.

Determining ideal cutoffs for defect dimensions has been a debate topic for years. Recent consensus suggests a depth of 2 mm as a threshold indicating a CSD [11]. Our study aligns with this consensus and introduces the same cutoff for depth in TVU. Furthermore, the study found that width and length also showed similar predictive values to depth, with 2 and 3 mm as their respective ideal cutoffs. These findings contribute to establishing standardized diagnostic criteria for CSD.

Although administering saline or gel as contrast agents enhances the diagnostic capabilities of 2D-ultrasound, [7, 19] it carries the risk of ascending infection and may lead to the overestimation of insignificant defects [7, 20]. Three-dimensional ultrasound, while showing promising diagnostic accuracy in the literature, requires further assessment of its costs and benefits compared to conventional 2D ultrasound [21, 22].

Scar defects enlarge and become easier to diagnose with each subsequent cesarean Sects. [10, 18, 23]. Our study supports this by achieving a significant positive correlation between the number of previous CSs and the CSD diagnosis. On the other hand, women with more previous CSs are typically older, making it challenging to assess age as an independent variable. While other studies have achieved an insignificant correlation between age and the presence of CSD, [24] this study revealed a negative correlation between them. This suggests that younger women with the same number of previous CSs may be more prone to scar defects. This may originate from the more robust inflammatory response and myometrial tension among younger participants. Yet, further investigation is needed to fully understand this correlation.

Inconsistencies in the literature regarding CSD diagnosis can be attributed to different assessment modalities, operator-dependent techniques, lack of conclusive diagnostic criteria, and varying study designs among diverse populations [25]. Therefore, multicenter studies with comprehensive data and standardized diagnostic features are warranted to establish more conclusive and generalizable findings.

To the point of our knowledge, this study is the first to design a diagnostic model for CSD, integrating patient history and TVU findings. By considering both aspects, the study offers a comprehensive approach that enhances CSD diagnosis accuracy. Furthermore, the relatively large sample size compared to previous similar studies contributes to the robustness of the findings and increases the reliability of the developed predictive model.

Despite its prevalence and known consequences, CSD remains a relatively novel and underrecognized pathology. Our study, aligning with Murji et al., underscores the importance of considering CSD in patients with AUB [5]. Through a straightforward ultrasound examination and medical history assessment, the identification or exclusion of CSD can avert unnecessary and costly diagnostic procedures like hysteroscopy. This not only streamlines the diagnostic process but also facilitates early detection, providing an opportunity to prevent adverse outcomes such as scar dehiscence or uterine rupture in case of pregnancy.

Given that AUB can serve as an indicator for gynecological malignancies like endometrial cancer, oncologists should be vigilant about CSD as a potential differential diagnosis. Scar dehiscence in individuals with CSD can manifest during procedures like Dilatation and Curettage (D&C), commonly employed for diagnosing endometrial malignancies [26]. Hence, recognizing the possibility of underlying CSD in patients with AUB can guide cancer diagnostic workups toward alternative methods, steering away from procedures that might pose risks in the presence of CSD.

Our results should be cautiously interpreted due to some inherent limitations. First, including only 100 patients referred to a single hospital because of AUB of unknown origin limits the generalizability of the study’s results to the entire population. It is imperative to acknowledge that defect sizes are smaller among asymptomatic individuals, potentially reducing the accuracy of the predictive model in this specific group [16]. Second, both hysteroscopy and TVU depend on operator skills. By allocating a single experienced operator to each procedure, we tried to minimize the relevant errors. Third, although hysteroscopy is considered the most accurate diagnostic modality for directly visualizing the intrauterine cavity, it is not an ideal gold standard and cannot achieve 100% accuracy in detecting CSD. Finally, saline administration during hysteroscopy may have inadvertently evacuated accumulated blood or excretions from the scar niche, leading to its shrinkage. This potential effect could hinder the precise diagnosis of CSD, impacting the study’s results. Therefore, our predictive model’s accuracy may increase when applied in regular clinical settings where this potential confounding factor is absent.

Consolidating the strengths and limitations of this study, we offer insights for future investigations to bolster the validity and comprehensiveness of findings. Enlarging sample sizes and broadening the geographical and ethnic diversity in subsequent studies will enhance the generalizability of results. Deploying machine learning or deep learning models on large datasets can help extract additional features from demographic data and images, culminating in the development of a robust first-line diagnostic tool [27]. For further exploration, we highly suggest including asymptomatic patients with CSD, exploring modalities beyond non-contrast TVU, and calculating reliability measures for operator-dependent techniques. These endeavors are of paramount importance in advancing research, providing a solid foundation for translating study outcomes into practical applications within clinical settings.

## Conclusions

Our study illustrates that combining variables such as age, the number of previous cesarean sections, and TVU-based defect length and width can effectively detect CSDs in 88% of participants. These findings have significant implications for evidence-based decision-making, leading to improved patient management and more efficient diagnostic technique utilization.

## Data Availability

The data are not publicly available due to privacy or ethical restrictions. The datasets generated and analyzed during the current study are not publicly available due to privacy or ethical restrictions but are available from the corresponding author upon reasonable request.

## Abbreviations

AUB: abnormal uterine bleeding
CS: cesarean section
CSD: cesarean scar defect
TVU: transvaginal ultrasound
